# Application of kernel functions for accurate similarity search in large chemical databases

**DOI:** 10.1186/1471-2105-11-S3-S8

**Published:** 2010-04-29

**Authors:** Xiaohong Wang, Jun Huan, Aaron Smalter, Gerald H Lushington

**Affiliations:** 1 School of Electrical Engineering and Computer Science University of Kansas, Lawrence, Kansas, 66045, USA; 2 Molecular Graphics and Modeling Laboratory,University of Kansas, Lawrence, Kansas, 66045, USA

## Abstract

**Background:**

Similaritysearch in chemical structure databases is an important problem with many applications in chemical genomics, drug design, and efficient chemical probe screening among others. It is widely believed that structure based methods provide an efficient way to do the query. Recently various graph kernel functions have been designed to capture the intrinsic similarity of graphs. Though successful in constructing accurate predictive and classification models, graph kernel functions can not be applied to large chemical compound database due to the high computational complexity and the difficulties in indexing similarity search for large databases.

**Results:**

To bridge graph kernel function and similarity search in chemical databases, we applied a novel kernel-based similarity measurement, developed in our team, to measure similarity of graph represented chemicals. In our method, we utilize a hash table to support new graph kernel function definition, efficient storage and fast search. We have applied our method, named G-hash, to large chemical databases. Our results show that the G-hash method achieves state-of-the-art performance for *k*-nearest neighbor (*k*-NN) classification. Moreover, the similarity measurement and the index structure is scalable to large chemical databases with smaller indexing size, and faster query processing time as compared to state-of-the-art indexing methods such as Daylight fingerprints, *C*-tree and GraphGrep.

**Conclusions:**

Efficient similarity query processing method for large chemical databases is challenging since we need to balance running time efficiency and similarity search accuracy. Our previous similarity search method, *G*-hash, provides a new way to perform similarity search in chemical databases. Experimental study validates the utility of *G*-hash in chemical databases.

## Introduction

Elucidate the roles of small organic molecules in biological systems, as studied in chemical genomics, is an emergent and challenging task. Traditionally the analysis of chemical genomics data was done mainly within pharmaceutical companies for therapeutics discovery, and it was estimated that only 1% of chemical information was in the public domains [1]. The landscape of public available chemical genomics data, however, has been changed dramatically in the last few years. With the Chemical Genetics Initiative and the Molecular Library Initiative (started by NIH in 2002, [2], and 2004, [3], respectively), publicly-available, digitalized data grow exponentially fast. The PubChem database, just to name an example, contains structures of more than 18 million chemical compounds [4]. With the rapid growth of public chemical databases, fast similarity search in large chemical databases has started to attract intensive research attentions. There are two approaches for similarity search for 2D or 3D structure of biomolecues. Most 3D structure based approaches compare three-dimensional shapes using a range of molecular descriptors [5][6]. Such methods provide fast query processing in large chemical databases but relatively poor accuracy since such methods may lost much of the structure information during compressing the three-dimensional shapes. In 2D based similarity search, we focus on the 2D connectivity of chemical structures. Current 2D similarity measurements may be roughly divided into two categories. In the fragment based method, scientists embed chemical structures in a high dimensional feature space, e.g. through Daylight fingerprints [7] with the Tanimoto distance [8]. Fragment based similarity measurement is by far the most widely used method and is adopted as the default choice in databases such as PubChem [4]. Graph based similarity measurements, in contrast, do not break the chemical structures into fragment and has started to gain popularity. In graph based method, we utilize graph to model chemical structures and utilize different graph similarity measurements such as the largest common subgraph approach [9], graph editing distance [10] or graph alignment algorithms [11] to measure the similarity of chemical structures [10]. Though graph methods have been successfully applied in cheminformatics research, as evaluated in our experimental study, none of them has achieved the goal of fast and effective similarity search in chemical databases, i.e. having computational efficiency in scaling to large chemical databases and computational efficiency in capturing the intrinsic similarity of graphs. With the fast growing of chemical databases, fast, effective, and indexable approaches are needed.

Our goal in this paper is to bridge the gap between graph kernel functions and similarity search for efficient and accurate similarity search in large chemical databases by applying our previous method, named *G*-hash [12]. In our method, we model a chemical structure by its two dimensional (2D) connectivity graph where nodes represent atoms and edges represent chemical bounds between atoms. We extract local features for each node and their neighboring nodes in the graphs. Using a hash table, a graph kernel function is defined to capture the intrinsic similarity of graphs and for fast similarity query processing. Our experimental results show that the *G*-hash method achieves state-of-the-art performance for similarity search in chemical databases. The retrieved* k* nearest neighbors by *G*-hash are more likely similar to the query chemical compared with the state-of-the-art indexing methods such as Daylight fingerprints and *C*-tree. Most importantly, the similarity measurement and the index structure is scalable to large database with smaller indexing size, faster indexing construction time, and faster query processing time as compared favorably with other indexing methods.

The rest of the paper is organized as follows. In the Related Work section, we will give an overview of related work on subcomponent search and chemical similarity search. In the Background Section, we will introduce the concept of graphs and graph modeling of chemical structures. In the Methods section, we discuss the details of our algorithm including our index structure and kernel function. In the Results section, we show a comprehensive experimental study using our method and competing methods, and discuss the influence of feature sets. Finally, in the Conclusions section, we conclude with a few remarks on the study.

## Related work

In this section we discuss two types of related work, i.e. subcomponent search and chemical similarity search. We work exclusively on the 2D connectivity graph of chemical structures and treat the following terms interchangeable: graphs and chemical structures, nodes and atoms, edges and chemical bounds.

### Subcomponent search

Many of the recent subcomponent(subgraph) search methods adopt a similar framework, decomposing chemical structures into a set of smaller pieces, treating each piece as a descriptor, and building a descriptor-based index structure for subgraph query. The most well-known algorithm in this category is the Daylight fingerprints [7]. In Daylight fingerprints, all paths up to a fixed length (e.g. 7) are retrieved as descriptors. A molecule is represented as a bit-string, indexed by the descriptors. Similar approach of using paths as descriptors is adopted by* GraphGrep*[13]. Though paths are easy to retrieve and easy to work with, the simplicity of paths limits their power in capturing the intrinsic similarity of chemicals.

Recognizing the limitation of paths,* gIndex*[14],* FG-Index*[15] and* GDIndex*[16] build indices using general subgraphs. GDIndex also incorporated a hash table of subgraphs for fast subgraph isomophism lookup. The main drawback of subcomponent search is that no quantitative similarity measurement is provided which makes it difficult to rank the search results in a meaningful manner.

### Chemical similarity search

Extending subcomponent strategy to similarity search in large compound databases is non-trivial. The most widely used strategy is previously mentioned the Daylight fingerprints approach, which treats a chemical compound as a bit-string and use various similarity metric for bit-strings, such as the Taminoto index [17] to measure the similarity of chemicals. Though fast, Daylight fingerprints provides only a coarse measurement of the true similarity of chemicals since majority of the features (i.e. paths) may not contribute to the chemical activity of the compounds and there is no feature selection step in the Daylight system.

Beside fragment-based method, maximal common subgraph (MCS) [18] was also utilized in measuring the similarity of graphs. Several heuristic strategies [19], based on specific properties of chemical structures, were proposed to improve the efficiency of MCS-based similarity search algorithm. Recently, anew backtracking algorithm was presented to compute MCS in [9]. Although such method shows better accuracy, the MCS computation still time-consuming.

In addition,* graph edit distance* and graph alignment [11] were also used in cheminformatics to measure graph similarity. Unfortunately, there is no easy way to index both measurements for large chemical structure databases.

## Background

Before we proceed to discuss the algorithmic details, we present some general background materials which include the introduction of the concept of* graphs* and chemical structures as graphs.

### Graphs

A* labeled graph G* is described by a finite set of nodes* V* and a finite set of edges* E* ⊂* V* × *V*. In most applications, a graph is labeled, where labels draw from a label set *λ*. A labeling function* λ*:* V* ∪* E* → Σ assigns labels to nodes and edges. For the label set Σ we do not assume any structure of Σ now; it may be a field, a vector space, or simply a set. Following convention, we denote a graph as a quadruple* G =* (*V, E,* Σ*, λ*) with aforementioned* V, E,* Σ, *λ,.* A graph *G =* (*V, E,* Σ,* λ* ) is a* subgraph* of another graph* G′* =(*V′*, *E′*, Σ′, *λ′*), denoted by* G* ⊆ *G′*, if there exists a 1-1 mapping *f *:* V*→* V′* such that

• for all* v ∈ V*, *λ*(*v*) =* λ′* (*f*(*v*))

• for all (*u, v*) *∈E*, (*f* (*u*), *f* (*v*)) *E*′

• for all (*u, v*) *∈E*, *λ* (*u, v*) = *λ′* (*f* (*u*), *f*(*v*))

### Graph modeling of chemical structures

**Figure 1:**
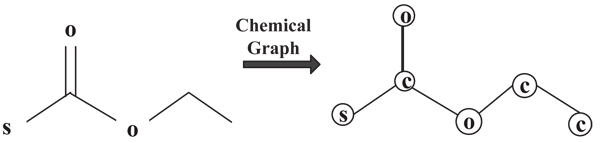
**Sample chemical structure and its graph representation.Left:** the sample chemical structure. **Right:** Graph representation of the sample chemical structure.

Chemical compounds are organic molecules that are commonly modeled by graphs. In our study, we adopt the 2D connectivity map where we use* nodes* in a graph to model* atoms* in a chemical structure and* edges* to model chemical *bonds* in the chemical structure. In the representation, nodes are labeled with the atom element type, and edges are labeled with the bond type (single, double, and aromatic bond). The edges in the graph are undirected, since there is no directionality associated with chemical bonds.* Figure *1 shows one sample chemical structure and its graph representation.

## Methods

Here we investigate the utility of graph kernel for chemical similarity measurement. Towards that end, we first give a overview of *G*-hash. We then briefly outline a graph kernel [12], which we will use, to define similarity of chemical structures. In particular, below we introduce details of the feature extractiion process, the index structure for fast similarity query and the kernel function for similarity measurement.

### Algorithm overview

**Figure 2:**
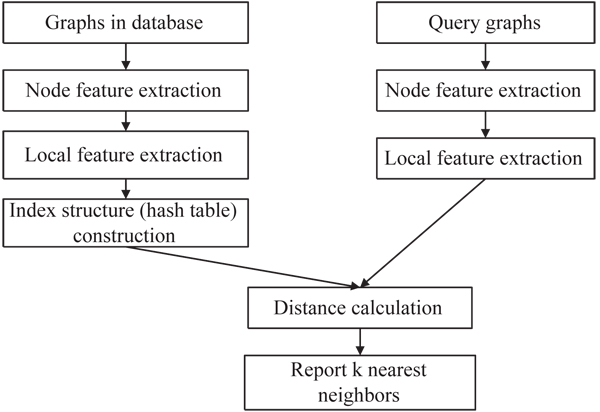
Flowchart of *G*-hash algorithm.

The flowchart of* G*-hash is show in* Figure *2. For graphs in database, we first extract node features with a graph wavelet analysis (details discussed later). We then build a hash table to store such features. For query graphs, we perform the same preprocessing techniques. Based on the hash table, we calculate distances between query graph and graphs in the database. Finally, top* k* nearest neighbors are reported.

In particular, the application of *G*-hash to chemical databases follows the below steps. In index construction, we utilize the following steps:

• For each chemical in the chemical database, extract node features for each atom in the chemical

• Using graph wavelet analysis, extract local features for each atom in the chemical 

• Discretize the combined features and hash the atoms in a hash table.

In the query processing phase, we utilize the following steps:

• For the query chemical, extract node and local features for each atom in the chemical

• Discretize the combined features and hash the atoms in a hash table using the same procedure in index construction

• Compute distances of the query chemical to the rest of chemicals

• Report the* k* nearest neighbors.

### Node feature extraction

To derive an efficient algorithm scalable to large graphs, our idea is to use a function Γ:* V* → ℝ*^ n^* to map nodes in a graph represented a chemical compound to a* n*-dimensional feature space that captures not only the node label information but also the neighborhood topological information around the node. Two steps involve this process: first *node feature extraction* through which we extract features associated with a node, and second* local feature extraction* through which we extract features in a local region centered at the specific node.

We use the following node (atom) features: atomic number, the histogram of atom types of immediate neighbor of the node, the local functional group information, and the histogram of the (immediate) chemical bond information. The atom type of the node is a single number. For histogram of neighboring atom types, we collect information for C, N, O, S, and group the rest atom types to "others" to save space. We have a total of five numbers in the histogram. For local functional group information, we collect whether the node is in part of a 5-node ring, a 6-node ring, a high-order ring, a branch, or a path, as did in [20]. We have a single number for this feature. For the histogram of the (immediate) chemical bond information, we have three numbers corresponding to single, double, and aromatic bonds. In the previously mentioned node extraction method, we ignore the neighborhood topology information of the chemical compound by focusing on atom physical and chemical properties. To add neighborhood topology information, we utilize a technique called the graph wavelet analysis, as originally presented in [21]. The output of the wavelet analysis is a vector of local feature averages, with the size of the vector controlled by a diffusion parameter *d*. Further details of the analysis can be found in [21].

### Structure matching kernel

With the feature extraction methods, we designed a structure kernel, specified below, to measure the similarity of graphs:(1)

**Figure 3:**
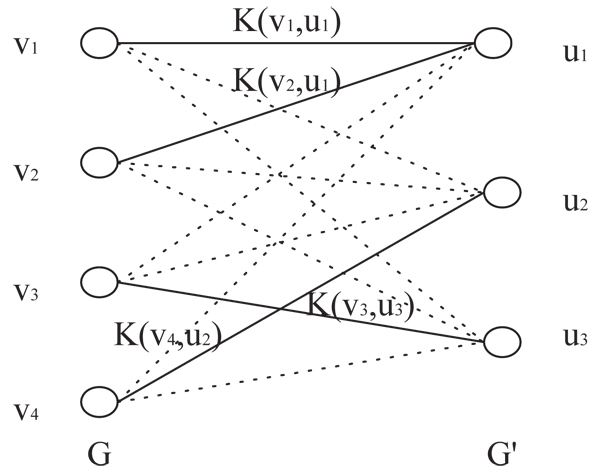
**A schematic representation of the structure matching kernel.**Highlighted edges (*v*1, *u*1),(*v*2, *u*1), (*v*3, *u*3),(*v*4, *u*2) have larger weights than the rest of the edges dashed.

*K* can be any kernel function defined in the co-domain of Γ. We call this function* K_m_* a* structure matching kernel.* We visualize the kernel function by constructing a weighted complete bipartite graph: connecting every node pair (u,v) *∈ V*[*G*] × * V*[*G*′] with an edge weighted by *K*(Γ(*v*), Γ(*v*)). In* Figure *3, we show a weighted complete bipartite graph for* V*[*G*] = {*v*_1_, *v*_2_, *v*_3_,* v*_4_} and* V*[*G*′] = {*u*_1;_*u*_2_,*u*_3_}.

From the figure we see that if two nodes are quite dissimilar, the weight of the related edge is small. Since dissimilar node pairs usually outnumber similar node pairs, in our design, we use the RBF kernel function, as specified below, to penalize dissimilar node pairs.(2)

where ||*X*|| _2_ is the* L*_2_ norm of the vector* X* .

### Similarity search with hash functions

To support effectively indexing, here we use a hash table where the key is the related node feature vector and the value is the node. Two chemicals are* similar,* if they share a lot of nodes that are hashed to the same cell since each node is represented by a feature vector which contains the local atomic and topological information. Since node features and local features may contain numeric value, we discretize each feature vector and map the feature value to an integer. After discretization, we hash all nodes in a chemical to the related hash table. We show an example of such hash table below.

**Figure 4:**
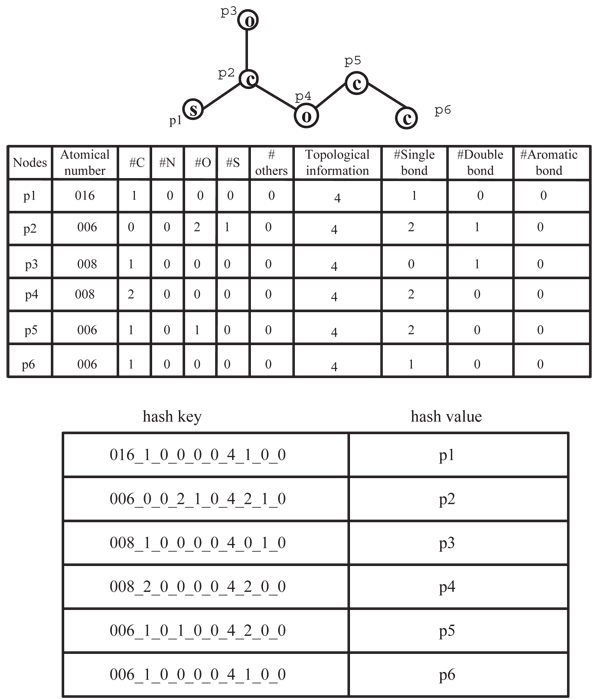
An example graph, related feature vectors, and the hash table contents.

**Example 1*** For simplicity, we apply the hash process to the single graph shown in Figure *1* whose nodes are numbered with p*1,* p*2,* p*3,* p*4,* p*5,* p*6 *shown in Figure *4.* We assume d=0 and each node has 10features. For example, the feature vector for node with the label of 'S' is [016,1,0,0,0,0,4,1,0,0] since its atomic number is '16'; it has only one neighbor with node label 'C', zero neighbor with node label 'N', zero neighbor with node label of 'O', zero neighbor with node label of 'S', and zero neighbor with node label of other atom symbol; it is in a path; and it connects with the neighbor through only one single bond. The feature vectors for all nodes are also shown in the Figure *4* and the sample hash table is shown in the bottom panel of Figure *4.

With the feature vector discretization and hash table, we revise the structure matching kernel using an hash-based approximation as described below since only similar nodes are involved into the kernel calculation and *K*(Γ*^h^*(*u*), Γ*^h^*(*v*)) ≈ 1 if RBF kernel is used.(3)

where* simi*(*v*) is the set containing the nodes from graph* G* that are hashed to the same cell as the node* v* does. |*simi*(*v*)| is the number of nodes in the set of* simi*(*v*). In other words, we only count the number of common nodes, belonging to the graph* G* and* G'* in this version.

Finally we compute the distances between the query chemical and chemicals in a chemical database to obtain the* k* nearest neighbors of the query chemical. The idea is to compute the Euclidian distance of two objects between their embeddings in the related Hilbert space according to the kernel function.

## Results

### Experimental setup

We have performed a comprehensive evaluation of our method by evaluating the classification effectiveness and scalability for large chemical databases. We have compared our method with other similarity measurements including the Daylight fingerprints [7], Wavelet Alignment Kernel [21], *C*-tree [10], GraphGrep [22], gIndex [23]. For *G*-hash, we extract 30 features for each node. We used the OpenBabel software package to compute Daylight Fingerprints [7] and *k*-nearest neighbors. For WA, we set the diameter* d =* 2 and use* haar* wavelet function. For *C*-tree, GraphGrep and gIndex, we use default parameters. All methods, except *C*-tree, were implemented using the C++ programming language and compiled using g++ with -O3 optimization.* C*-tree was developed in Java and compiled using SUN JDK1.5.0. We performed our experiments on a Linux cluster where each node has a dual-core Intel Xeon EM64T 3.2GHz processor and 4G memory running CentOS 4.

### Data sets

We chose a number data sets for our experiments. The first five data sets are established data taken from Jorisson/Gilson Data Sets [24]. The next seven data sets are manually extracted from BindingDB data sets [25]. The last one is NCI/NIH AIDS Antiviral Screen data set [26]. For the Jorissen data sets, there are five proteins for which 100 chemical structures are selected with 50 chemical structures clearly bind to the protein(called "active" ones) and the other 50 ones similar to the active ones but clearly not bind to the target protein. See [24] for the further details. For the BindingDB database, we manually selected 7 proteins with a wide range of known interacting chemicals (ranging from tens to several hundreds). For the purpose of classification, we convert the real-valued binding activity measurements to binary class labels. This is accomplished by dividing the data set into two equal parts according to the median activity value (we also deleted compounds whose activity value is equal to zero). Table 1 shows the characteristics of the data sets. In the same table, positive compounds refer to those with higher activity values or binding to the target protein and negative compounds refer to those with lower activity values or not binding to the target protein.

**Table 1:** Data set characteristics.

data set	#S	#P	#N	#V	#E
PDE5	100	50	50	44.7	47.2
CDK2	100	50	50	38.4	40.6
COX2	100	50	50	37.7	39.6
FXa	100	50	50	45.75	48.03
AIA	100	50	50	48.33	50.61
AChE	183	94	89	29.1	32.0
ALF	151	61	60	23.8	25.2
EGF-R	497	250	247	24.6	27.1
HIV-P	267	135	132	43.0	46.2
HSP90	109	55	54	29.84	32.44
MAPK	336	168	168	28.0	31.1
HIV-RT	482	241	241	22.18	24.39

#S: total number of compounds, #P: number of positive compounds, #N: number of negative compounds, #V: average number of nodes, #E: average number of edges.

We use the NCI/NIH AIDS Antiviral Screen data set, which contains 42,390 chemical compounds retrieved from DTP's Drug Information System, as a large chemical database. There is a total 63 types of atoms in this data set; the most frequent ones are C, O, N and S. The data set contains three types of bonds: single-bond, double-bond and aromatic-bond. We randomly sampled 1000 chemicals as the query data set.

### Similarity measurement evaluation with classification

We have compared classification accuracy using *k*-NN classifier on the 12 Jorissen data sets and BindingDB data sets with different similarity measurements. For the WA method, we first obtain kernel matrix, and then calculate distance matrix to obtain the* k* nearest neighbors. For subgraph indexing methods such as gIndex and Graphgrep, we sketch one way to use them for similarity search. This method contains three steps: (i) randomly sample subgraphs from a query, (ii) use those subgraphs as features and compute the occurrences of the subgraphs in graph databases, and (iii) search for nearest neighbors in the obtained feature space. Clearly, the accuracy depends on the number of features. Here we pick 20 features for gIndex. We use standard 5-fold cross validation to obtain classification accuracy. We have tested different* k* values ranging from 3 to 11 in classifications. The quality of the results are similar and we only report results with* k =* 5.

**Figure 5:**
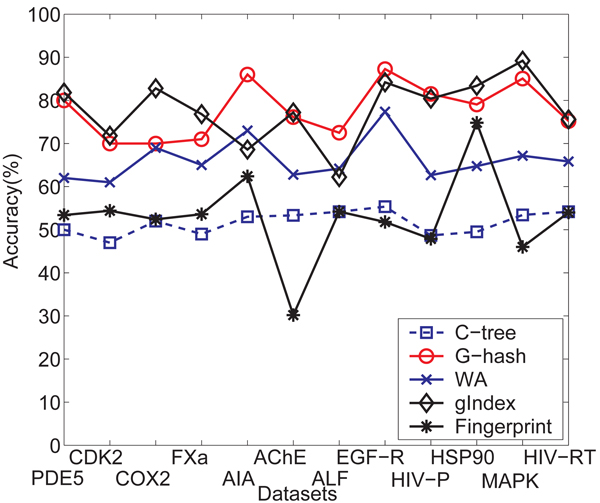
Comparison of averaged classification accuracy over cross validation trials.

The accuracy of the classification is shown in* Figure *5. The average precision and recall are shown in* Table *2 and *Table *3 respectively. The accuracy results statistical information is shown in* Table *4. From* Figure *5, we know that *C*-tree and Daylight fingerprints show the worst performance. Theyjust are a little better than the random guess. WA method is better than them since it similarity measurement is based on kernel function. gIndex based similarity measurement and *G*-hash has similar classification performance with about 78% of average accuracy and outperform others. *G*-hash outperforms *C*-tree and Daylight fingerprints on all twelve data sets, with at least 18% improvement on most of data sets. The average accuracy difference between *G*-hash and *C*-tree and Daylight fingerprints are around 23% and 22% respectively. The precision of *C*-tree and Daylight fingerprints are lower than 50% for almost all data sets.* G*-hash is comparable to gIndex on precision and recall, too.

**Table 2:** Average Precision for different data sets.

dataset	*G*-hash	WA	*C*-tree	Fingerprint	gIndex
PDE5	95.48	83.16	31.20	53.02	96.00*
CDK2	79.87	73.81	51.82	57.23	87.81*
COX2	92.40*	75.88	54.85	51.62	82.00
FXa	96.93*	95.78	29.36	52.80	93.23
AIA	96	98.93	36	64.61	99.00*
AChE	77	66.46	62.63	27.80	79.62*
ALF	77.38	72.14	32.59	55.61	82.88*
EGF-R	88.62	72.75	55.41	52.42	96.40*
HIV-P	83.64	56.9	40.81	46.99	95.22*
HSP90	85.66	58.19	48.72	76.57	93.00*
MAPK	84.01	66.31	53.25	44.40	95.79*
HIV-RT	80.93	69.38	54.11	54.20	84.61*

Asterisk (*) denotes the best precision for the data sets among *G*-hash, WA, *C*-tree, Daylight fingerprint and gIndex methods.

**Table 3:** Average recall for different data sets.

Dataset	*G*-hash	WA	*C*-tree	Fingerprint	gIndex
PDE5	73.19	58.06	46.93	58.80	73.60*
CDK2	67.17	55.87	46.70	47.20	67.82*
COX2	64.22	63.57	51.46	54.21	87.01*
FXa	64.63	58.23	42.06	55.62	71.19*
AIA	80.70*	64.81	55.33	54.41	60.20
AChE	76.13	63.63	44.15	27.40	85.20*
ALF	69.84	61.25	53.83	75.98*	65.00
EGF-R	86.22*	79.64	55.81	54.00	81.61
HIV-P	80.44*	63.4	47.62	42.20	75.40
HSP90	76.31	63.4	47.62	71.00	91.38*
MAPK	86.83*	70.52	72.16	42.01	85.79
HIV-RT	72.83	67.78	56.78	60.80	73.40*

Asterisk (*) denotes the best recall for the data sets among *G*-hash, WA, *C*-tree ,Daylight fingerprint and gIndex methods.

**Table 4:** Accuracy results statistical information for G-hash, C-tree WA, gIndex and Daylight fingerprint on all data sets.

method	*G*-hash	*C*-tree	WA	gIndex	Fingerprint
average	77.81	51.64	66.23	77.83	5292
derivation	6.29	2.68	4.83	7.51	10.03

**Figure 6:**
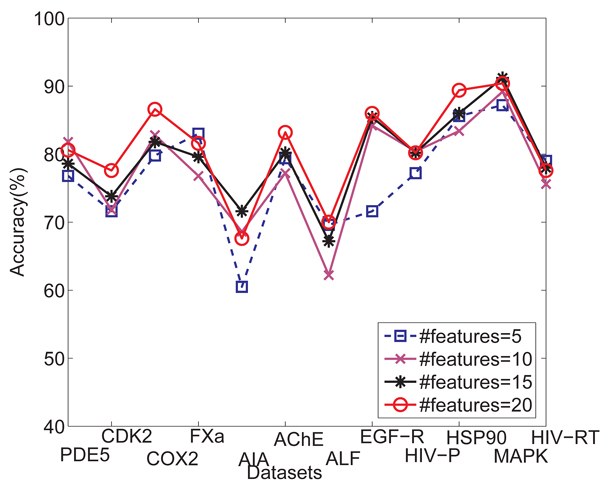
Comparison of averaged classification accuracy over cross validation trials when different feature sets are used for gIndex method.

Here we use 20 features for gIndex. As we mentioned before, the performance of gIndex depends on the use of feature set.* Figure *6 shows the accuracy comparison among different feature sets with 5 features, 10 features,15 features and 20 features for the method of gIndex. From* Figure *6, we know that more features provide better classification performance than less features do. Here, 20 feature set provides the best classification results, 15 features set and 10 features set have a middle performance, 5 features set has the worst performance.

### Chemical enrichment study

**Figure 7:**
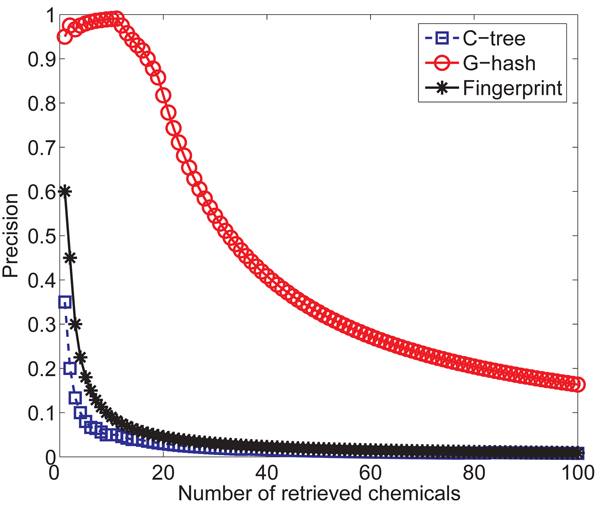
Average "precision" curves over 20 queries.

In this section, we perform the enrichment study of* G*-hash, Daylight fingerprints and* C*-tree. Towards this end, we randomly picked 20 chemical compounds from 110 inhibitors of focal adhesion kinase 1 (FADK 1) with AID810 from PubChem [4]. We call those 20 chemicals as test data set. We augment this test data set to the NCI/NIH AIDS Antiviral Screen data set to form a new database. Then we pick one chemical from these 20 chemicals as the query chemical to search the new database and retrieve 100 nearest neighbors. According to these 100 results, we calculate the "precision" curve. Specifically, for the top* k* similarity compound, we compute* precision* as the percentage of chemicals in the top* k* compounds belongs to the true 19 hits and plot the change of precision along with the number of retrieved chemicals. Obviously, the high precision shows good performance. After repeating the above steps for 20 times, we calculate the average precision curve shown in* Figure *7. Although Daylight fingerprints performs better than *C*-tree, both of them show the low precision. *G*-hash performs much better than Daylight fingerprints and *C*-tree. From* Figure *7, we see that the precision is about 0.85 when the total number of retrieved chemicals is equal to 19 for *G*-hash which means that 16 chemicals in the test data are contained in the top 19 nearest neighbors of the query chemical. The result is as same as what we expected. Edit distance based similarity measurement used by* C*-tree prefers large graphs. For Daylight fingerprints, two graphs sharing more small substructures or patterns are considered to be similar. But as we all know, the connection or position of these substructures also determines the similarity of graphs. Our method,* G*-hash, not only consider the number of common small substructures but also consider the connection between them through using features reflecting local topological information and chemical information.

### Robustness

**Figure 8:**
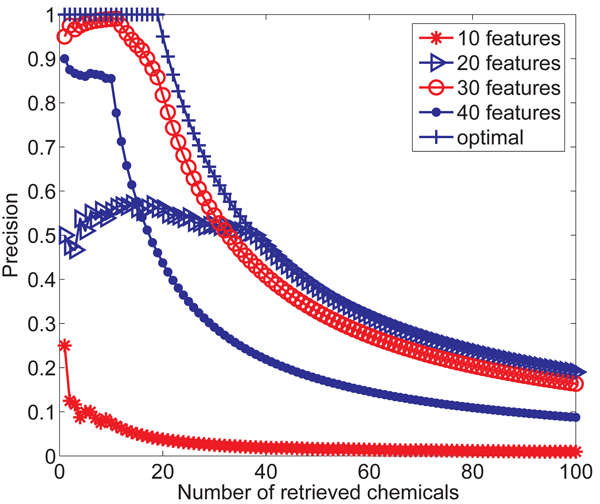
Average precision curves over 20 queries for *G*-hash with different feature sets.

In this section, we evaluate the robustness of *G*-hash by using four different feature sets for the enrichment study mentioned above. In the first set of features, we use 10 features as discussed in the subsection of Node Feature Extraction. For other three data set, we use wavelet analysis to extract features from the local region centered at the particular node. We use* d =* 1 with 10 additional features,* d =* 2 with 20 additional features and* d =* 3 with 30 additional features. So we have 4 feature sets with sizes 10, 20, 30 and 40. The average precision curves over 20 queries and the optimal precision curve are shown in* Figure *8. We draw the optimal precision curve in this way: if the number is retrieved chemicals is less than 19, the precision is equal to 1; otherwise, the precision is equal to 19 over the number of retrieved chemicals. *G*-hash with 10 features shows the worst performance which is similar to that of *C*-tree and Daylight fingerprints shown in* Figure *7. *G*-hash with 20 features has a much improvement. *G*-hash with 30 features gives the best performance which is near to the optimal performance.* G*-hash with 40 features has a little worse performance than *G*-hash with 30 features. With less features, more nodes pairs are hashed into the same cell. This case prefers those graphs sharing too many small subcomponents. With more features, just a few nodes pairs are hashed into the same cell. This case prefers those small graphs. Therefore the distance between graphs can not accurately represent their similarity with too large or small feature sets.

### Scalability

**Figure 9:**
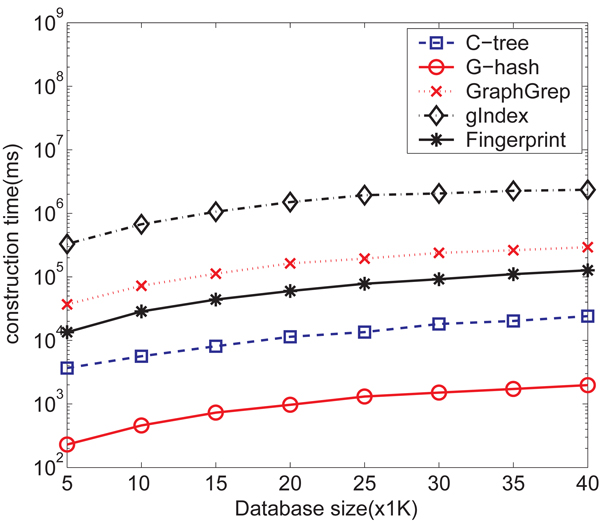
Index construction time comparison for NCI/NIH AIDS data set on *G*-hash, *C*-tree, GraphGrep, gIndex and Daylight fingerprints.

**Index Construction** We compare index size and average index construction time for different methods. Towards that end, we have sampled different number of graphs ranging from 5,000 to 40,000.* Figure *9 shows the index construction time in milliseconds with respect to the size of database for* G*-hash,* C*-tree, GraphGrep, gIndex and Daylight fingerprints. The construction time for *G*-hash is much faster than those for other four methods with a speed-up up to three order of magnitudes.

**Figure 10:**
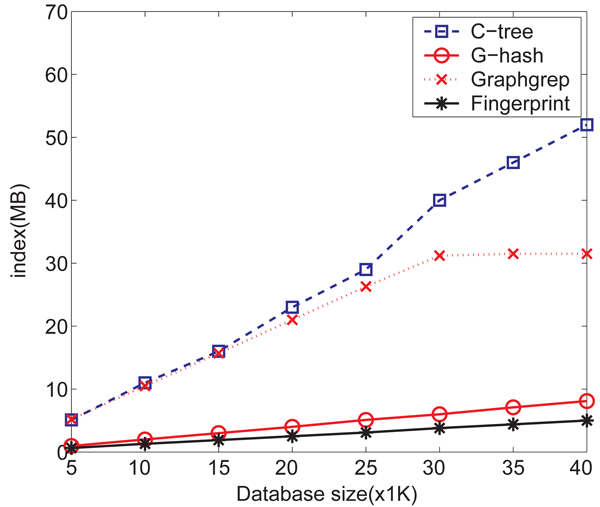
Index size comparison for NCI/NIH AIDS data set on *G*-hash, *C*-tree, GraphGrep and Daylight fingerprints.

*Figure *10 shows the sizes of constructed indexes with respect to database sizes. The index size of *G*-hash grows slowly with increasing database size while that of *C*-tree increases sharply. Daylight fingerprints shows a very similar scalability to that of *G*-hash. A sharp index size increase is observed for the methods of *C*-tree and GraphGrep. We did not show the index size of gindex since the size is much larger than the rest of the methods. For example gIndex takes 7.2 MB index spaces for 5,000 chemicals and 20.9MB for 10,000 chemicals.

**Figure 11:**
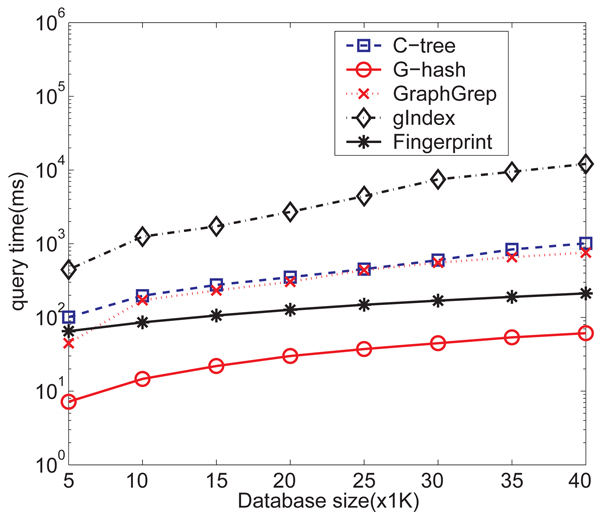
Query time comparison for NCI/NIH AIDS data set on *G*-hash, *C*-tree, GraphGrep, gIndex and Daylight fingerprints.

**Query Processing Time** There is no direct way that we could compare* G*-hash and subgraph indexing methods such as gIndex and Graphgrep and we use the way that we outlined before (random sample a few subgraphs from the query graph and then perform subgraph query search). Clearly the overall query processing time depends on the many subgraphs we sample. To estimate the lower bound of the overall query processing time, we randomly sample a SINGLE (one) subgraph from each of the 1000 querying graph and use subgraph indexing method to search for the occurrence of the subgraph. We record the average query processing time for each query. This query processing time is clearly the lower bound since we use only one subgraph from the query graph.* Figure *11 shows the average query time (over 1000 randomly samples query chemicals) of different index methods in milliseconds with respect to the size of database. gIndex is the worst one. Fingerprints do the query faster than *C*-tree and GraphGrep which are comparable. *G*-hash is the fastest one. When the size of database increases, *G*-hash scales better than Daylight Fingerprints, with around an order of magnitude speedup.* G*-hash performs better than* C*-tree, with two orders of magnitude speedup.

### Discussion

**Feature set influences** One of the key factors for determining both the accuracy and efficiency of the *G*-Hash method is the feature extraction methods r that maps nodes to a high-dimensional feature space. In order to evaluate the results, we have compare five sets of features. In the first set of features, we use two features(atom type and another feature from wavelet analysis with* d =* 1) as discussed in the Methods section. In the second set, we use 10 features described in the subsection of Node Feature Extraction. In the third feature set, we dropped the immediate chemical bond information from the first set and obtained seven features. In addition, we use wavelet analysis to extract features from the local region centered at the particular node. We use* d =* 1 with 10 additional features and *d =* 2 with 20 additional features. So we have 5 feature sets with sizes 2,7, 10, 20, and 30.

**Figure 12:**
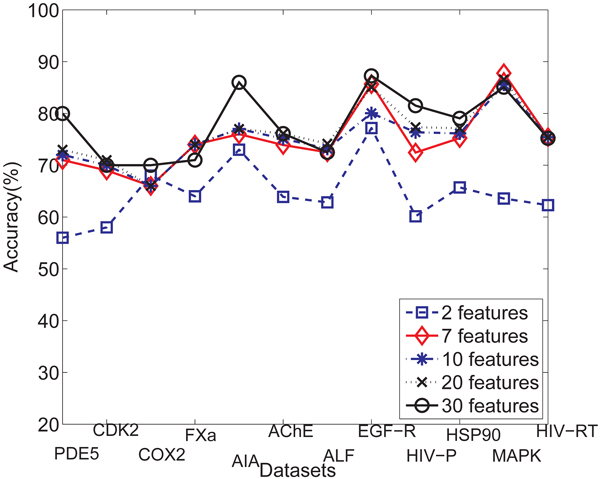
Average accuracy for different feature sets.

We have tested the classification accuracy with different feature sets. The average accuracy on 12 datasets is shown in *Figure *12. When more features are used, we can obtain better results. The largest difference happens between 2 features and 7 features which means that the histogram of atom types of immediate neighbors and the local functional group information make a big contribution to improve classification performance. Another relatively large difference happens between 20 features and 30 features which means the topological information of neighbors with hop distance equal to 2 much more makes sense. The difference between 7 features and 10 features is very small which shows that the histogram of the immediate chemical bond information plays little role in improving classification accuracy. We also tested the query processing time difference between different feature sets shown in* Table *5. Both too less features and too more features will speed up query processing. With too less features, many modes are likely hashed into the same cell so that the hash table is too short and less query processing time is needed. With more features, nodes are more likely to be hashed to different cells. If too more features are used so that the nodes of the query graph just are hashed into a few cells and hence we could speed up query processing time.

**Table 5:** Average query running time for different number of features with different database sizes.

# of features	Average Running Time (ms)	
	**10k**	**20k**	**30k**	**40k**

2	30.97	67.71	102.9	139.39
7	109.12	219.2	333.86	451.96
10	107.01	233.13	355.69	471.22
20	23.3	56.6	80.76	113.74
30	14.67	29.94	44.71	61.16

So to obtain both good classification performance and fast query processing, relatively more features should be used. In our case, the feature set with 30 features is the best choice.

## Conclusions

In summary, similarity search plays a critical role in cheminformatics. Efficient similarity query processing method for large chemical databases is challenging since we need to balance running time efficiency and similarity search accuracy. Here we applied our previous similarity search method,* G*-hash, combining hash based indexing and graph kernel function, and applied it into the similarity search in the large chemical databases. The key features of* G*-hash are that the *k*-NN query time is scalable to large databases and has better classification accuracy. We have compared our method with commonly used methods such as Daylight fingerprints [7] and C-tree [10] and have demonstrated the utility of our method.

## Competing interests

The authors declare that they have no competing interests. 

## Authors contributions

XW developed methods, implemented the software, and drafted the manuscript. JH offered the research idea, participated in the discussion during research, and wrote part of the paper. AS was involved in testing the data set and helped revise the manuscript. GL provided advices on the chemical aspect of the work.

## References

[B1] Dobson C Chemical space and biology. Nature 2004 432 7019 824 8 10.1038/nature03192 15602547

[B2] Tolliday N Clemons PA Ferraiolo P Koehler AN Lewis TA Li X Schreiber SL Gerhard DS Eliasof S Small Molecules, Big Players: the National Cancer Institute's Initiative for Chemical Genetics. Cancer Research 2006 66 8935 42 10.1158/0008-5472.CAN-06-2552 16982730

[B3] Austin C Brady L Insel T Collins F NIH Molecular Libraries Initiative. Science 2004 306 5699 1138 9 10.1126/science.1105511 15542455

[B4] PubChem. Http://pubchem.ncbi.nlm.nih.gov

[B5] Ballester PJ Richards WG Ultrafast shape recognition for similarity search in molecular databases. Proceedings of the ROYAL SOCIETYA 2007

[B6] Rush TS Grant JA Mosyak L Nicholls A A shape-based 3-D scaffold hopping method and its application to a bacterial protein-protein interaction. J 2005 48 1489 1495 10.1021/jm040163o 15743191

[B7] Daylight Fingerprints. 2008 Software available at http://www.daylight.com

[B8] Girke T Cheng LC Raikhel N ChemMine. A Compound Mining Database for Chemical Genomics. Plant Physiology 2005 138 573 577 15955920 10.1104/pp.105.062687 PMC1150377

[B9] Cao Y Jiang T Girke T A maximum common substructure-based algorithm for searching and predicting drug-like compounds. Bioinformatics 2008 24 13 i366 74 18586736 10.1093/bioinformatics/btn186 PMC2718661

[B10] He H Singh AK Closure-tree: an index structure for graph queries. Proc. International Conference on Data Engineering'06 (ICDE) 2006

[B11] Vert JP The optimal assignment kernel is not positive definite. Tech. Rep. HAL-00218278, French Center for Computational Biology 2008

[B12] Wang XH Smalter A Huan J Lushington GH G-hash: towards fast kernel-based similarity search in large graph databases. Proc 2009 472 480 10.1145/1516360.1516416 PMC2860326 20428322

[B13] Giugno R Shasha D GraphGrep: a fast and universal method for querying Graphs. Proceedings of the International Conference in Pattern Recoginition(ICPR) 2002

[B14] Yan X Yu PS Han J Graph indexing: a frequent structure-based approach. SIGMOD 2004

[B15] Cheng H Yan X Han J Hsu CW Discriminative Frequent Pattern Analysis for Effective Classification. Proceedings of the 23rd IEEE International Conference on Data Engineering (ICDE) 2007

[B16] Williams D Huan J Wang W Graph Database Indexing Using Structured Graph Decomposition. Proceedings of the 23rd IEEE International Conference on Data Engineering (ICDE) 2007

[B17] Jacob L Hoffmann B Stoven V Vert JP Virtual screening of GPCRs: an in silico chemogenomics approach. Tech. Rep. HAL-00220396, French Center for Computational Biology 2008 10.1186/1471-2105-9-363 PMC2553090 18775075

[B18] Cone MM Venkataraghavan R McLafferty FW Molecular Structure Comparison Program for the Indentification of Maximal Common Substructures. J 1977 99 7668 7671 10.1021/ja00465a041

[B19] J Raymond ea Heuristics for similarity searching of chemical graphs using a maximum common edge subgraph algorithm. J 2002 42 305 316 11911700 10.1021/ci010381f

[B20] Cheng J Yu JX Ding B Yu PS Wang H Fast Graph Pattern Matching. 23rd International Conference on Data Engineering 2008

[B21] Smalter A Huan J Lushington G Graph Wavelet Alignment Kernels for Drug Virtual Screening. Proceedings of the 7th Annual International Conference on Computational Systems Bioinformatics 2008 19642292

[B22] Shasha D Wang JTL Giugno R Algorithmics and Applications of Tree and Graph Searching. Proceeding of the ACM Symposium on Principles of Database Systems (PODS) 2002

[B23] Yan X Yu PS Han J Graph Indexing Based on Discriminative Frequent Structure Analysis. ACM Transactions on Database Systems (TODS) 2005

[B24] Jorissen R Gilson M Virtual Screening of Molecular Databases Using a Support Vector Machine. J. Chem. Inf. Model 2005 45 3 549 561 10.1021/ci049641u 15921445

[B25] Liu T Lin Y Wen X Jorrisen RN Gilson M BindingDB: a web-accessible database of experimentally determined protein-ligand binding affinities. Nucleic Acids Research 2007 35 D198 D201 17145705 10.1093/nar/gkl999 PMC1751547

[B26] NCI/NIH AIDS Antiviral Screen data set. 1999 set available at http://dtp.nci.nih.gov

